# Travel grants and how to use them (when there's no travel)

**DOI:** 10.1242/dmm.046987

**Published:** 2020-09-21

**Authors:** Annabel Nicholson, Julija Hmeljak

**Affiliations:** The Company of Biologists, Bidder Building, Station Road, Histon, Cambridge CB24 9LF, UK

## Abstract

**Summary:** This Editorial discusses how DMM and its publisher, The Company of Biologists, have adapted the financial support they provide to the biological community in these unprecedented times.

**Figure:**
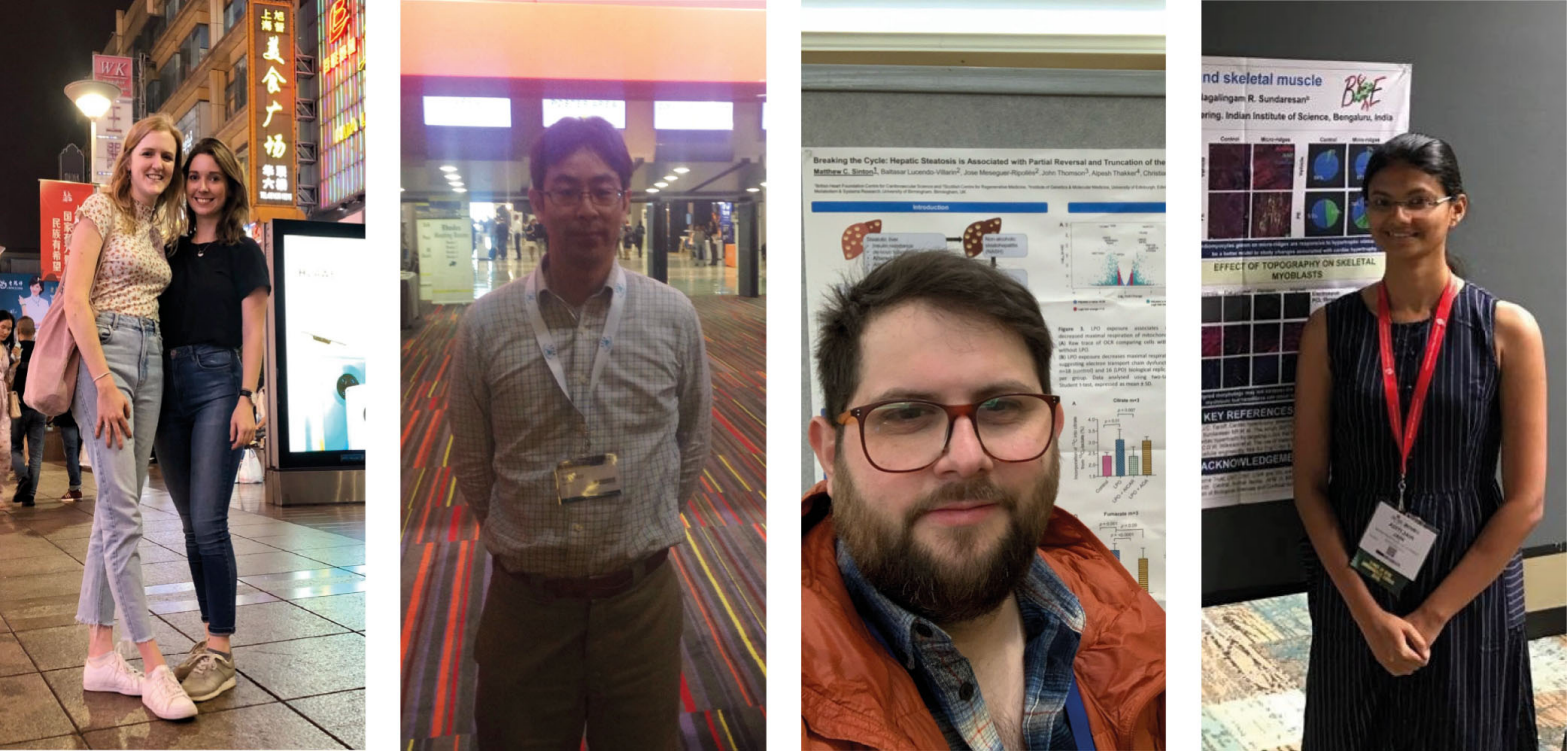
**A few recent DMM Conference Travel Grant recipients enjoying their conferences.**

As part of The Company of Biologists' family of scientific journals, Disease Models & Mechanisms (DMM) is committed to supporting the biomedical community. The Company of Biologists is a not-for-profit publisher, using any surplus it generates to benefit biology and the biological community. This takes the form of organising Scientific Workshops and Journal Meetings, which unite biologists across different fields, as well as funding several scientific societies, providing meeting grants and awarding Travelling Fellowships that help send early-career researchers to international laboratories for collaborative visits.

DMM is also a strong advocate for early-career researchers' active participation in conferences. The international stage is vital in determining future career directions for young scientists, with conferences and meetings opening up collaborations, networking and presenting opportunities. To support the future generations of researchers, DMM launched Conference Travel Grants in 2015. These offer financial support to early-career researchers wishing to present their work at scientific meetings that fit within DMM's scope. There are four funding rounds a year to accommodate the busy conference calendars and give applicants flexibility.

Over the past 5 years, we have awarded 234 Conference Travel Grants to applicants from around the world. To build a truly global scientific community, these grants have no geographic, citizenship or residency restrictions for applicants and there are no limits on the destination country.

The reports our grantees submit after they have returned from their conferences give us important insight into what junior scientists value most. For Takeshi Ninchoji from Uppsala University, Sweden, the 2019 European Association for Vision and Eye Research (EVER) meeting in Nice, France was an opportunity to expand his way of thinking. “I learned and found not only knowledge but also the way of thinking in ophthalmology. It means that I also learned research strategy in this field,” said Takeshi. “I have met a number of academics and professionals from different countries who have similar research interests.”

Erin Hedges from King's College London, UK, attended the 30th International Symposium on ALS/MND (amyotrophic lateral sclerosis/motor neurone disease), which was held in Perth, Australia in December last year. For Erin, attending the Symposium had a meaningful impact on her methodologies. “I decided to alter some of my experiments based on a couple of talks I attended, and I hope this will result in more meaningful and impactful results from my experiments,” she said. She also gained confidence from talking to other delegates. “For much of my project, I have been optimising and using devices and techniques that were not previously used in my lab. Seeing other groups and more senior scientists presenting impressive data using similar techniques gave me confidence in my approach.”

Refining techniques and learning new ones is another important step in a researcher's journey. As well as funding conferences, DMM also offers Travelling Fellowships. These grants enable graduate students and postdoctoral researchers to make collaborative visits to other laboratories and establish lasting collaborations. Grantees have the opportunity to share their experiences with other members of our community.

It goes without saying that the world is looking a little different this year and we have adjusted our support in response. The global scientific community has shown remarkable resilience during the COVID-19 pandemic, which has threatened to cut many collaborative and networking ties. Conference organisers have adapted quickly, migrating planned events to online platforms. DMM wants to continue its support for PhD students and postdocs during this particularly tumultuous time, which is why we have expanded the types of meetings that are eligible for Conference Travel Grant funding to include virtual conferences. Although networking and discussions look different in the virtual conference ecosystem, we strongly encourage early-career members of our community to register for these meetings and to apply for our upcoming grants and other financial support. Now, more than ever, early-career researchers need help to stay connected with their peers and senior colleagues.

Before the pandemic reared its head, the academic world was already assessing the merits of virtual conferencing. The personal interactions at conferences are vital, but they aren't always the greenest, quickest, most accessible or most targeted ways of interacting as a scientific community. COVID-19 took the long-term planning away from us and the whole world transitioned online in unison. It's been a steep learning curve, and, as well as figuring out what works, we've also come a long way in learning what definitely doesn't. Over the past few months, there has been a cultural shift towards a more open community willing to share ideas. We are staying connected with colleagues online, but we now need to experiment with different formats to meet new people with the data and skills that will push the research on.

The Company of Biologists is committed to this ongoing discussion. We recruited a Sustainable Conferencing Officer, and, later this year, the Company will host its first virtual Journal Meeting and Workshop. What we learn this year by going digital will create new opportunities to share research locally and across the globe. These experiences and expertise will help us tailor our funding to fit the needs of the junior members of our community even better.

2020 may be a year of imaginary travel across Zoom backgrounds, but the science is still happening and we're not planning on missing it. Importantly, we remain committed to helping you do the same.

